# The therapeutic potential of Wild Bitter Melon to ameliorate muscle atrophy in a murine model

**DOI:** 10.22038/AJP.2024.24011

**Published:** 2024

**Authors:** Sima Seifi, Seyedeh Elnaz Nazari, Amir Avan, Nima Khalili-tanha, Fatemeh Babaei, Saman Soleimanpour, Fereshteh Asgharzadeh, Mousa-al-reza Hajzadeh, Majid Khazaei, Abdoljalal Marjani

**Affiliations:** 1 *Metabolic Disorders Research Center, Department of Biochemistry and Biophysics, Golestan University of Medical Sciences Gorgan, Golestan, Iran*; 2 *Metabolic syndrome Research center, Mashhad University of Medical Sciences, Mashhad, Iran*; 3 *Department of Medical Physiology, Faculty of Medicine, Mashhad University of Medical Sciences, Mashhad, Iran*; 4 *Basic Sciences Research Institute, Mashhad University of Medical Sciences, Mashhad, Iran*; 5 *Medical Genetics Research center, Mashhad University of Medical Sciences, Mashhad, Iran*; † Equal first author

**Keywords:** Wild bitter melon, Atrophy, Inflammation, Oxidative stress

## Abstract

**Objective:**

Muscle atrophy due to immobility is a common complication of many diseases and a consequence of therapeutic processes. Immobility and inactivity have been shown to be associated with increased inflammation. The aim of this study was to investigate the therapeutic potential of Wild Bitter Melon (WBM) (Momordica charantia Linn) on muscle atrophy due to immobility in a mouse model.

**Materials and Methods:**

This study was performed in two phases of atrophy and recovery on male BALB/c mice which were divided into 3 groups: control, immobilized, and experimental. The treatment period with WBM at a dose of 400 mg/kg daily by gavage was 17 days, including 7 days of being immobilized and 10 days of recovery. At the end of each phase, half of the mice from each group were examined regarding the four limb grip strength, and then histological and biochemical analyses were done.

**Results:**

The tissue level of malondialdehyde (MDA) oxidative stress index in the atrophy phase in the atrophy group (5.4567±0.522) nmol/g compared to the control group (3.455±0.065) nmol significantly (p 0.001) <) increased. Also, the tissue level of MDA in the WBM group (3.87±0.035) showed a significant decrease compared to the atrophy group (p<0.01). The strength percentage of four limbs in the mice of the treatment group (-23.46±2.45) was significantly higher than that of the atrophy group (-30.60±3.15) at the end of the atrophy phase.

**Conclusion:**

The results suggest that the use of WBM reduces the degree of inflammation, oxidative stress and muscle damage, as well as muscle atrophy, which may improve the muscle atrophy in mice.

## Introduction

Muscle atrophy due to immobility is a common complication of many diseases (e.g. strokes and chronic diseases) and treatment processes (e.g. immobilization of limbs in orthopedic surgery and tendon repair) (Cohen et al., 2015; Rommersbach et al., 2020). Immobility is one of the most important factors leading to atrophy of the mid-leg muscles (Rommersbach et al., 2020). Due to the prevalence of inactivity and weight intolerance in the lower extremities, several studies have been performed on the muscles of the lower extremities and they have shown that the soleus and gastrocnemius muscles have the highest and fastest rate of atrophy during inactivity and weight intolerance in the lower extremities (Bodine, 2013; Gao et al., 2018; Psatha et al., 2012).

Muscle fibers can be divided into two main types: slow muscle fibers type (I) and fast muscle fibers type (II). Muscle resorption can happen through several distinct signaling pathways with different sensitivities between two fiber subtypes of skeletal muscle. Type I muscle fiber atrophy is reduced by exercise and weight bearing (Garrett et al., 1983). With continued immobilization, type II muscle fibers also become atrophic. Unlike atrophy of type I muscle fibers, this atrophy cannot be reduced or prevented with resistance training. Considering that muscle atrophy occurs due to inactivity due to atrophy of muscle fibers type I and II and type II muscle fiber atrophy cannot be prevented or treated with light exercise, it seems that the continuation of muscle atrophy is more related to the atrophy of type II muscle fibers (Wang and Pessin, 2013). Therefore, finding a way to prevent or reduce the rate of atrophy during immobility and sedentary lifestyle can help to maintain muscle mass and, consequently, physical function. It has been found that immobility and inactivity are associated with increased inflammation. The expression of inflammatory cytokines can be related to the progression of atrophy. Immobility-induced inflammation can activate an atrophic program by activating the transcription factor NF-κB (Van’t Klooster et al., 2020). During chronic inflammation, one of the mechanisms that cause muscle atrophy is increased oxidative stress (Powers, 2014a). NF-κB activation associated with increased reactive oxygen species (ROS) production plays a vital role in increasing the expression of pro-inflammatory cytokines such as TNF-α. Then, pro-inflammatory cytokines may stimulate the migration and infiltration of inflammatory cells and the secondary wave of ROS production, further amplifying the inflammatory cascade and damage (Xin et al., 2016). One of the strategies to reduce the occurrence and to prevent this problem includes reducing inflammatory reactions and oxidative stress. 

Natural products such as medicinal herbs and their extracts have always been used to treat various diseases (Patwardhan et al., 2005). One of these plant products is Wild Bitter Melon (WBM) (*Momordica charantia* Linn), which is part of the Cucurbitaceous family (Kwatra et al., 2013a; Kwatra et al., 2013b; Nkambo et al., 2013) and is used in Asia and Europe to treat various pathological conditions (Chao et al., 2011). So far, several studies have been conducted on the biological activities of WBM, including blood sugar lowering, antiviral, antibacterial, antitumor, antioxidant, immune modulation, antidiabetic, antimutagenic, anthelmintic, anti-lipidemia activities (Chao et al., 2011). These properties are due to the complex chemical composition of WBM, which includes terpenoids, tannins, carbohydrates, saponins, resins, flavonoids, sterols, anthraquinones, phlobatannins , glycosides, fatty acids, amino acids, and phenolic compounds (de Oliveira et al., 2018). This study evaluated the therapeutic effect of WBM extract as a potent antioxidant in improving muscle atrophy due to immobility in mice.

## Materials and Methods

**Materials**

 The capsules containing WBM extract powder (one capsule containing 600 mg dry weight) were bought from a local pharmacy store produced by AmerMed Co. (Philadelphia, USA). Male BALB/c mice aged 7 to 8 weeks were bought from the animal facility of Mashhad University of Medical Sciences, Mashhad, Iran. Standard laboratory conditions (12-hr light cycle and 12-hr dark cycle, and unrestricted use of water and standard diet) were provided. This experiment was performed on 3 groups of 12 mice (mice were randomly divided into these groups). Experiments on animals in this project were completed according to the guidelines approved by the ethics committee of Golestan University of Medical Sciences with the ethics ID IR.GOUMS.AEC.1401.014.

**Animal studies**

This study was performed in two phases atrophy and recovery. The treatment period was 17 days, including 7 days of being immobilized in one limb then on the 8th day, the mice splints were opened, and 10 days for recovery. Thirty-six mice were randomly assigned into three groups, which are listed below: 

Control or healthy group (n=12): atrophy phase (n=6) and recovery phase (n=6), Immobility group (immobility model) (n=12): atrophy phase (n=6) and recovery phase (n=6), WBM-treated group (immobility model, treated with 400 mg/kg WBM extract powder) (n=12) : atrophy phase (n=6) and recovery phase ( n=6) (Ma et al., 2017). 

**Figure 1:**
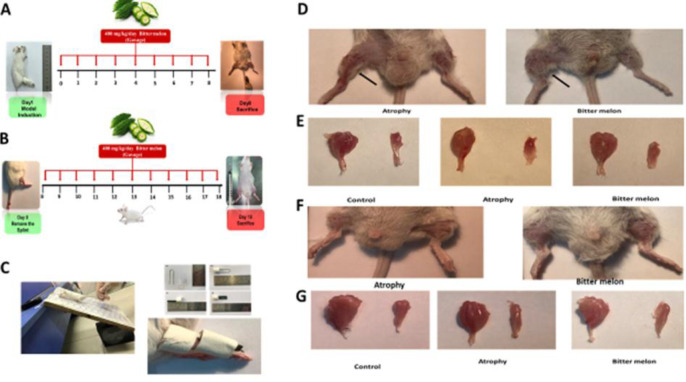
Study Design and Muscles Macroscopic Pictures of model system in two phases of atrophy and recovery. A-C) The treatment period was 17 days, including 7 days of immobility in one limb (A), then on the eighth day, the mice's splints were opened and 10 days were considered for recovery (B). The tools used to model immobility and atrophy (C). Image of a mouse subjected to unilateral hindlimb immobilization (D). (D-F) Atrophy is characterized by the loss of muscle tissue, the leg muscle of mice atrophied in two groups of atrophy and WBM in two stages of atrophy and recovery is indicated by a black arrow, respectively. (E and G) The picture of the gastrocnemius and soleus muscles in the three studied groups at the end of the atrophy and recovery phase, respectively (E) and (G).

To create a model of immobility and atrophy, the method used in previous studies was used. In short, with very simple and accessible tools, a splint was prepared for the animal and the animal's right leg was immobilized in a position where the knee was in the extension position and the ankle was in the plantar flexion position (Figure 1) (You et al., 2015). On day 0, all mice were weighed, and limb strength was measured using a force meter which will be thoroughly explained.

**Atrophy phase**

The animals then received normal water and food for 7 days, and in addition, WBM -treated group was treated daily with 400 mg/kg WBM powder dissolved in distilled water and administered by gavage (Figure 1). On the 8th day, the mice splints were opened, and the following investigations were performed on half of the mice in each group (Lang et al.,2012).

**Recovery phase**

 The remaining 6 mice in each group recovered for 10 days (after opening splint) and during which time treatment was continued in the WBM-treated group. Then, after 10 days, the muscle tissues of gastrocnemius and soleus were separated and all the analyses performed in the atrophy phase were also performed in this phase (Figure 1) (Lang et al., 2012).

**Measure four-limb grip strength**

 On the eighth day, half of the animals in all three groups were examined by a force gauge for muscle strength. In this regard, we used a thin wire mesh connected to a digital force meter (DS2-110 500 N, Toyohashi, Japan). The mice were held by their tails and gently moved on the mesh; as the mice grip it with their paws, the force is transmitted to the force meter; the assessment was repeated three times. A single operator performed this procedure to reduce variability (Figure 1).

**Weighing of gastrocnemius and soleus muscles**

 To examine the leg muscle tissue for atrophy, the mice were sacrificed and then the gastrocnemius and soleus muscle tissue were removed, photos of these muscles were taken, and the tissue was immediately weighed using the 0.001 g accurate electronic digital weighing scale. (SF-400 10 Kg Beijing, China). This procedure was done fast, so as not to waste or make any errors due to dehydration. The muscle tissues were weighed both separately and then together. The target muscle tissues of each mouse were normalized against body weight. 

**Histological studies**

For the histological analysis, tissue samples were kept in 4% formaldehyde neutralized buffer and embedded in paraffin wax. Afterward, the slides were prepared at a thickness of 4 µm using a cryotome (Shandon, Pittsburgh, PA 15275, California, USA). Light microscopy was used to examine the sections, which were stained with Hematoxylin-Eosin (H&E) stain for evaluation of muscle fiber size and cross-section area. The muscle cross-section and fiber size were quantified using NIH Image software (Image J) (Fang et al., 2021).

**Measurement of malondialdehyde (MDA) level**

PBS (phosphate buffered solution with pH 7.4) was used to homogenize the ~50 mg tissues. The homogenates were centrifuged for ten minutes and the supernatants were evaluated for malondialdehyde (MDA; an oxidative marker). Then, 15 g of trichloroacetic acid (TCA) was mixed with 0.375 g of thiobarbituric acid (TBA) and 2 ml of concentrated hydrochloride, and reached a final volume of 100 ml with distilled water. Then 2 ml of the obtained solution was mixed with 1 ml of homogeneous tissue and put in a boiling water bath for 50 min. Following cooling of the mixture, 25 μl of concentrated HCl solution was added to the tube and centrifuged at 1000 g for 10 min. After discarding the supernatant, the optical density (OD) of the mixture was read against blank at 535 nm. Tissue MDA content was computed by the following equation (where C (M): concentration in molar, A: optical density) C (M) = A / 1.56 * 105 (Janero, 1990).

**Molecular studies**

**Extraction of RNA and quantitative real-time PCR**

Real- time PCR was used to evaluate the effect of WBM powder on the mRNA expression level of inflammatory cytokines, interleukin 6 (IL-6) and TNF- α. Total RNA extraction was performed using the total RNA extraction kit according to the manufacturer's instructions from all groups (Pars Tos, Mashhad).cDNA synthesis kit (Pars Toos, Mashhad) was used for cDNA synthesis. The gene expression levels were measured using SYBR Ampliqon Green Moster Mix kit by ABI applied bio system. The thermal program consisted of 1 cycle of 95°C for 15 min, followed by 30-40 cycles including 95°C for 30 sec and 60-55°C for 60 sec. A comparative method was used to analyze the mRNA level normalized against GAPDH (2 -∆∆Ct) (Haddad et al., 2005).

**Biochemical analysis**

Inflammatory cytokines, TNF-α and IL-6 levels, and nitrite (NO2-) levels were assessed in tissue of all groups using specific sandwich ELISA according to the manufacturer's kit (ZellBio GmbH, Ulm, Germany). The level of inflammatory interleukins was measured in the tissue homogenate. To prepare the muscle tissue for this test, they were homogenized and then centrifuged at ambient temperature and at 10000 rpm for 10 min. To evaluate the level of muscle damage, the serum troponin I level was measured using a specific sandwich ELISA according to the manufacturer's kit (ZellBio GmbH, Ulm, Germany).

**Statistical analysis**

Data are presented as mean±standard deviation (SD) and were analyzed by the one-way analysis of variance (ANOVA). It should be noted that the mice used in our experiment were in the homogenous body weight range, but to reduce even the slightest differences that could affect our evaluation, we normalized some of our results by dividing the outcomes by the body weights. In order to compare groups, we used LSD post hoc test.

## Results

**Macroscopic observation**

After 7 days, the splint was removed, and the affected limb was observed macroscopically, and pictures were taken. The atrophied muscle in the atrophy group mice was significantly smaller than the atrophied muscle in the WBM-treated group mice (Figure 1). Also, after 10 days of opening the splint, at the end of the recovery phase, the atrophied muscle was examined again macroscopically, and the atrophied muscle in the atrophy group was comparatively smaller than the atrophied muscle in the group treated with WBM (Figure 1).

**Muscle weight**

**Muscle weight in the atrophy phase**

**Figure 2:**
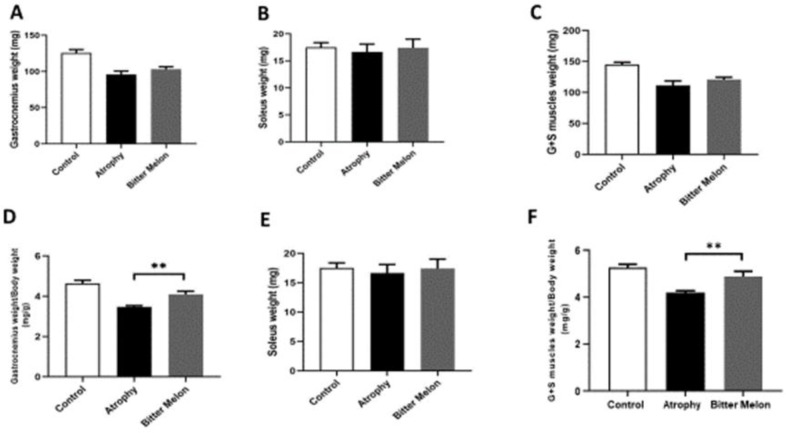
Muscles weight in the atrophy phase. The mean gastrocnemius muscle of mice from all three groups of control, atrophy, and treatment with WBM was compared (A). The mean soleus muscle was compared in the three groups (B). The weight of total of both muscles from all 3 groups was compared (C). The weight of gastrocnemius muscle of each mouse was normalized to the total body weight, which showed a significant increase in the muscle mass of the WBM-treated group compared to the muscle weight of the atrophic group (p<0.01) (D). The weight of the soleus muscle of each mouse was normalized to the total body weight and no significant difference between the groups was observed (E). The total weight of both muscles was normalized to the total body weight and showed a significant difference between the WBM-treated group and the atrophic group (p<0.01) (F)

Our results showed that WBM reduced muscle atrophy caused due to immobility in the WBM-treated group compared with the atrophy group. After sacrificing the atrophic phase mice, the gastrocnemius and soleus muscles were removed from all three groups on the 8th day. The weight of each muscle was examined separately and also together. Also, to normalize the muscle weight, the total body weight of each mouse was determined. As shown in Figure 2, the weight of gastrocnemius muscle of mice from all three groups of control (125.71±4.61 mg), atrophy (95.87±4.75 mg), and treatment with WBM (103.00±3.51 mg) was compared. Figure 2 compares the weight of soleus muscle in the three groups. In Figure 2, the weight of total of both muscles from all 3 groups are compared. Figure 2 shows that the weight of gastrocnemius muscle for each mouse was normalized to the total body weight and showed a significant increase in the muscle mass of the WBM -treated group (4.10±0.15 mg) compared to the muscle weight of the atrophic group (3.47±0.06 mg) (p<0.01). In Figure 2, no significant difference was observed between the weights of soleus muscle of each mouse normalized to the total body weight. In Figure 2, the total weight of both muscles was normalized to the total body weight and showed a significant difference between the WBM-treated group (4.860±0.220 mg) and the atrophic group (4.190±0.083 mg) (p<0.01).

**Muscle weight in the recovery phase**

**Figure 3:**
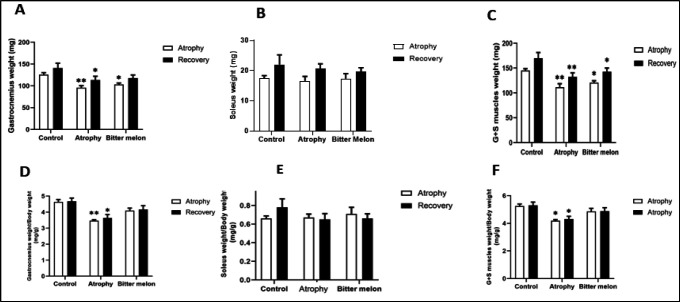
Weights of the gastrocnemius and soleus muscles in the recovery phase and the atrophy phase. The weight of the gastrocnemius muscle in each of the atrophy and recovery phases in the atrophy group and WBM-treated group was compared with the control group of each phase, which showed a significant decrease in the muscle mass of the atrophy group in the atrophy phase (p<0.01) and the atrophy group in the recovery phase (p<0.05), and the treated group in the atrophy phase (p<0.05) (A). The mean weight of the soleus muscle in each of the atrophy and recovery phases in the atrophy group and WBM-treated group were compared with the control group of each phase (B). The weight total of both muscles in each of the atrophy and recovery phases in the atrophy group and WBM-treated group was compared with the control group of each phase, which showed a significant decrease in the total weight of both muscles of the atrophy group in the atrophy phase (p<0.01) and the atrophy group in the recovery phase (p<0.01) (C). The weight of gastrocnemius muscle for each mouse was normalized to its total body weight, which showed a significant decrease in the muscle mass of the atrophic group compared to the muscle weight of the control group in each of the atrophy and recovery phases respectively (p<0.01), (p<0.05) (D). The weight of the soleus muscle for each mouse was normalized to the total body weight and no significant difference between the groups was observed (E). The total weight of both muscles was normalized to the total body weight and showed a significant difference when comparing the control group with the atrophic group in each of the atrophy and recovery phases (p<0.05) (p<0.05) (F)

Figure 3 shows a comparison of the weights of the gastrocnemius and soleus muscles of all three groups of mice in the recovery phase and the atrophy phase. There was no significant difference in the weight of these two muscles and the sum of these two muscles weight among the three groups in the recovery phase.

**Four-limb grip strength**

**Four-limb grip strength in the atrophy phase**

**Figure 4:**
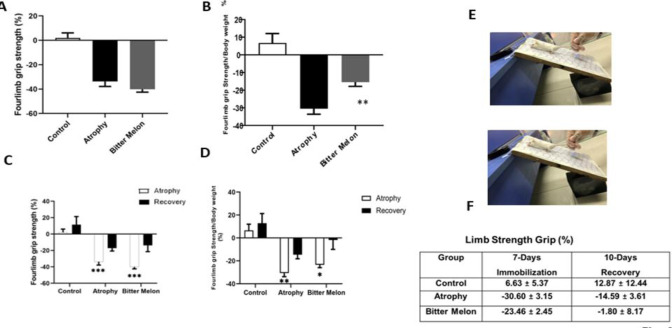
Four limb grip strength. Atrophy phase: the limb strength of the mice atrophic group and the mice treated with WBM group were calculated as a percentage compared to the limb strength of the control group (A). Average limb strength normalized to the weight of each mouse in all 3 study groups. There was a significant difference between the limb strength of the WBM -treated group (%- 23.46±2.45) compared to the limb strength of the atrophy group (%-30.60±3.15) (p<0.01) (B). Recovery phase: the results of the limb strength of the three groups of mice between the stages of atrophy and recovery (the star shows the comparison of each group with the control group((C). The results of the Limb strength normalized to weight in 3 groups and each group in two phases) the star shows the comparison of each group with the control group((D). Image of a thin wire mesh attached to a digital force gauge (DS2-110 500 N, Toyohashi, Japan) for measuring muscle strength in mice (E-F).

 This test was used to evaluate the muscle strength and see how the immobilization period and WBM treatment can affect the force generated by the mice. The limb strength of the atrophic group and the WBM group was calculated as a percentage compared to the limb strength of the control group (Figure 4). Then the limb strength of each mouse from each group was normalized by its body weight. The average normalized limb strength of each group is shown in Figure 4. There was a significant difference in the limb strength between the WBM -treated group (%- 23.46±2.45) and the atrophy group (%-30.60±3.15) (p<0.01).

**Four-limb grip strength in the recovery phase**

After removing the splint, during the recovery time, the muscles improved, and normal movement can improve rehabilitation; therefore, an increase in the results of grip strength of mice in the three studied groups was observed in the recovery phase compared to the atrophy phase. Figure 4 shows the mean strength percentage of the normalized four-limb with the weight in the control group (%12.87±8.44), atrophy group (%-14.59±3.61), and WBM-treated group (%-1.80±8.17). 

**Histological findings**

**Figure 5:**
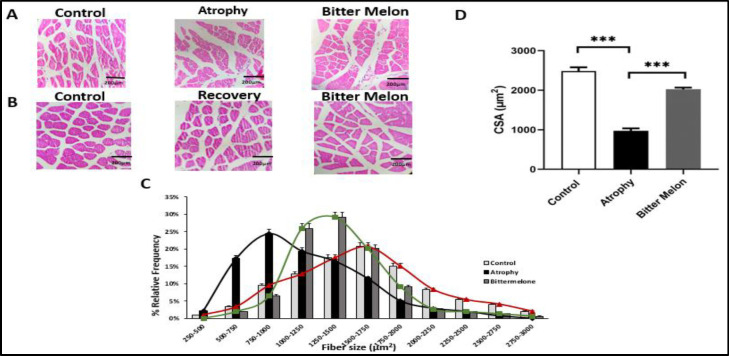
Histological examination of fiber size and cross-sectional area (CSA) (μm2) of fibers in response to atrophy and WBM treatment in both phases of the study. Image of muscle cross-section using NIH Image software (Image J) in the atrophy and recovery phases, respectively (Figure 5.A) (Figure 5.B) (10X). Relative abundance of fiber size in square micrometers (Figure 5.C). In figure 5.D results show that the size of fibers decreased in the atrophy group compared to the control group p<0.001), and the size of fibers increased in the WBM -treated group compared to the atrophy group (p<0.001).

To confirm the effect of WBM in reducing atrophy, we used H&E staining and Image J software to evaluate muscle cross-section and muscle fiber size in the three groups: control, atrophy and WBM. The muscle fiber sizes were reduced during the immobilization, and muscle bundles were scarcer in a microscope field of view (Figure 5). The size of fibers in the atrophy group (750-1000) µm² decreased significantly compared to the control group (1500-1750 µm²). WBM extract had a positive effect on muscle tissue, as the fiber size distribution shifted toward larger sizes. In Figure 5D, results show that the size of fibers decreased in the atrophy group compared to the control group (p<0.001), and the size of fibers increased more in the WBM-treated group (1250-1500 µm²) compared to the atrophy group (p<0.001) (Figure 5D). 

**Biochemical and molecular tests**

**Figure 6:**
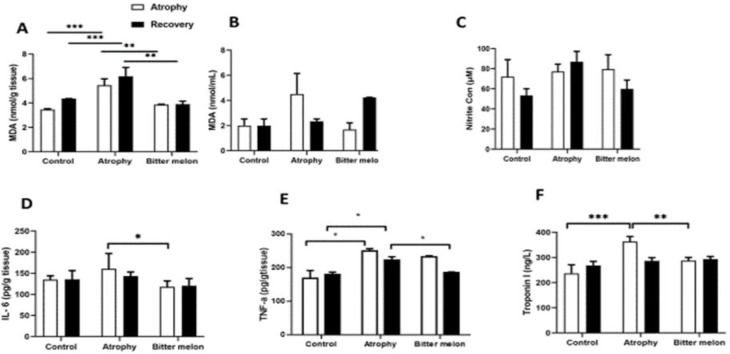
Oxidant and inflammatory markers and index of muscle damage: MDA level, an oxidant index, in muscle tissue (A) and in serum samples (B) of three study groups in two phases of atrophy and recovery. Nitrite concentration between three groups in both phases (C). The expression level of the inflammatory cytokine IL-6 at the protein level in the target muscle tissue in the 3 studied groups in the two phases of atrophy and recovery (D) The expression level of the inflammatory cytokine TNF-α at the protein level in the target muscle tissue in the 3 studied groups in the two phases of atrophy and recovery (E). Troponin I (TnI) serum level, index of muscle damage, in three study groups in two phases of atrophy and recovery (F) Statistical significance is represented as follows: p<0.05, p<0.01 and p<0.001.

The MDA levels, which is an oxidant marker, increased after immobilization not only in muscle tissues (Figure 6) but also in serum samples (Figure 6). Tissue MDA levels in the atrophy group (5.4567±0.522 nmol/g) increased significantly (p<0.001) in the atrophy phase compared to the control group (3.455±0.065 nmol/g). The results showed (Figure 6) that WBM in the atrophy phase significantly reduced the levels of MDA in the WBM group (3.87±0.035 nmol/g) compared to the atrophy group (p<0.001). These results showed that WBM exerts its protective effects by reducing oxidative stress reactions in atrophy. At the end of the recovery phase, the evaluation of oxidant stress showed that continued treatment with WBM in the WBM-treated group (3.9±0.25 nmol/g) significantly reduced MDA levels compared to the atrophy group (6.19±0.72 nmol/g) (p<0.01). Nitrite concentration was not significantly different among the three groups in both phases (Figure 6)

Tissue IL-6 levels in the control group (135±9.07 pg/g), in the atrophy group (160.22±36.85 pg/g) and in the WBM -treated group (118.08±14 p g /g) in atrophic muscle tissue in the phase atrophy are shown in Figure 6. The results showed that IL-6 levels in the group treated with WBM decreased compared to the atrophy group (p<0.05). These results suggest that the protective effects of WBM against atrophy after immobilization can be mediated at least in part by its anti-inflammatory responses in the mouse model.

TNF-α levels in the control group and in the atrophy group and the WBM-treated group in atrophic muscle tissue are shown in Figure 6. TNF-α levels in the atrophy group increased significantly (p<0.05) in both phases compared to the control group. The results showed that TNF-α levels in the group treated with WBM (182±0.25 pg/g) decreased compared to the atrophy group (223±0.82 pg/g) in the recovery phase (p<0.05). 

 The serum level of troponin I in the atrophy group increased significantly compared to the control group (p<0.001). This factor was significantly reduced in the group treated with WBM (287.49±12.23 ng/L) compared to the atrophy group (363.45±19.95 ng/L), which indicated less muscle damage in our treatment group (p<0.01). There was no significant difference in troponin I serum levels among the three study groups during the recovery phase (Figure 6). 

**Figure 7:**
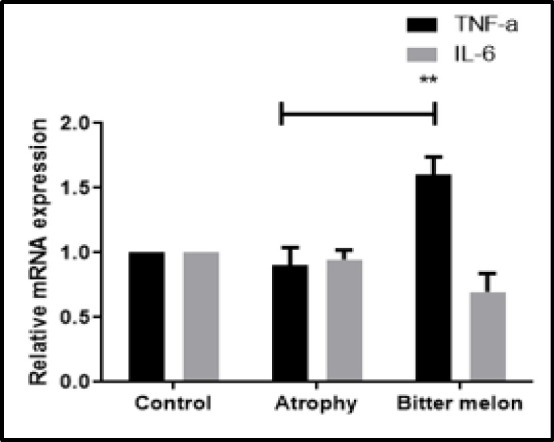
The expression level of the inflammatory cytokine: Comparison of the expression of inflammatory cytokines IL-6 and TNF-α at the mRNA level in two atrophy and WBM groups at the mRNA level in the target muscle tissue in the atrophy phase.

Also, the results of IL-6 expression at the mRNA level showed no significant difference between the treatment and atrophy groups in the atrophy phase (Figure 7).

The results of the expression of the inflammatory cytokine TNF-α at the mRNA level in the atrophy phase are shown in Figure 7. The results showed that the expression of the inflammatory factor TNF- α in the group treated with WBM significantly increased compared to the atrophy group (p<0.01) (Figure 7).

## Discussion

Inactivity-induced muscle atrophy results from both decreased protein synthesis and increased protein degradation. Although several factors are involved in regulating the rate of protein breakdown and synthesis in skeletal muscle, immobility and inactivity have been shown to be associated with increased inflammation in the same organ and systemic inflammation (Van’t Klooster et al., 2020). Stable expression of inflammatory cytokines is detrimental to muscle mass because it activates signal pathways that suppress protein synthesis and enhance protein breakdown that causes muscle cell atrophy (Reid and Molan, 2011). Inflammation due to immobility itself can lead to an atrophic program under the command of the transcription factor NF-κB (Judge et al., 2007). During chronic inflammation, it is believed that one of the main mechanisms that cause muscle atrophy is an increase in oxidative stress (Bonetto et al., 2012). Disruption of redox signaling, due to enhanced production of reactive oxygen species (ROS) and reduced antioxidant capacity, is an important regulator of signaling pathways that control both protein synthesis and proteolysis in skeletal muscles (Powers, 2014b). Prolonged inactivity of skeletal muscle due to immobility leads to a chronic increase in ROS production and consequent oxidative damage to these immobilized muscle fibers (Kondo, 1991). NF-κB activation associated with increased ROS production subsequently plays a critical role in the expression of pro-inflammatory cytokines such as TNF-α (Xin et al., 2016). These chemokines may facilitate the migration and infiltration of inflammatory cells and the secondary wave of ROS production and increase the inflammatory cascade and injury (Xin et al., 2016).

Because antioxidants can prevent oxidative stress caused by inactivity in skeletal muscle, treatment with antioxidants can potentially preserve protein synthesis and prevent accelerated proteolysis. In fact, multiple studies have shown that selected antioxidants (such as vitamin E) have the potential to reduce muscle atrophy due to inactivity of limb and respiratory muscles (Duarte and Soares, 1997; Levine et al., 2008; Servais et al., 2007). Contrary to these findings, several studies have concluded that the use of antioxidant supplements did not protect muscles against atrophy despite increasing antioxidant capacity in immobile muscles (Desaphy et al., 2010; Faridet al., 2005; Koesterer et al., 2002). Currently, the use of antioxidants as a therapeutic intervention to protect athletes and the patient population from immobile muscle atrophy is not widely accepted (Powers, 2014b). Therefore, more research is needed to demonstrate that antioxidant therapies are safe and effective in protecting against inactivity-induced muscle atrophy.

One of these plant products is Wild Bitter Melon (WBM) (*Momordica charantia* Linn) which is part of the Cucurbitaceae family (Kwatra et al., 2013a; Kwatra et al., 2013b; Nkambo et al., 2013) and is used in Asian and European countries to treat various pathological conditions. It has been reported that WBM has antioxidant, anti-inflammatory, anti-diabetic, anti-cancer, antihypertensive and antimicrobial activity (Chao et al., 2011). Numerous reports from WBM have demonstrated its potential antioxidant properties. The protective activity of WBM is believed to be mediated by increasing the activity of antioxidant enzymes and decreasing lipid peroxidation (Ching et al., 2011; Dhar et al., 2007; Sulaiman and Ooi, 2013; Thenmozhi and Subramanian, 2011). A study compared the antioxidant activity of different plants. The highest antioxidant activity was determined with WBM extract (Santos et al., 2010). Another study examined the antioxidant activity of WBM extract and found that it could improve levels of superoxide dismutase (SOD), catalase (CAT), glutathione-s-transferase and glutathione in various tissues (Tripathi and Chandra, 2010). The results of experimental studies have shown that different WBM extracts can have strong anti-inflammatory effects (Chuang et al., 2020; Huang et al., 2015; Kung et al., 2020). Examination of the antioxidant activity of the phenolic extract of WBM fruits showed that WBM extract had a dose-dependent protective effect on oxidative stress (Kumar et al., 2010). Also it was suggested that WBM fruit extract could reduce *Propionibacterium acne*-induced inflammation by inhibiting the release of pro-inflammatory factors (TNF-α, IL-8 and IL-1β) into target cell types (Hsu et al., 2012).

 In line with these results, our results showed that the tissue level of MDA in immobilized muscle was increased in the atrophy group compared to the control group, and there may be a correlation between atrophy and MDA levels. However, oral administration of WBM can decrease the tissue level of MDA. Also, based on the results of reducing the expression of pro-inflammatory cytokines in the WBM treatment group compared to the atrophy group, we hypothesized that WBM can act as an antioxidant and anti-inflammatory agent that can improve muscles. Another finding was the quadruped grip strength test, which cleared that during the period of immobility and non-use of the limb, the target limb is affected and loses its natural strength, resulting in less force. The results of this study showed that WBM administration increased grip strength, although this increase was not as strong as the control group. This effect was observed not only during immobilization but also during the recovery phase. Also, in histological studies on the size of fibers and the cross-sectional area of muscle tissue, it was found that the treatment with WBM significantly increased the fiber size and cross-sectional view in terms of the number of muscle fibers. Based on the findings of this study, the oral administration of WBM extract improves antioxidant and anti-inflammatory status by affecting the balance of oxidative stress, suppressing the expression of pro-inflammatory cytokines, and inhibiting penetration of inflammatory cells. Reducing oxidative stress and reducing inflammatory responses can reduce the serum level of troponin I, muscle damage, and muscle atrophy due to immobility. We have seen improved muscle atrophy by continuing treatment with this plant extract powder in the recovery phase. The results obtained from this study suggest that the use of WBM can be promising for reducing the atrophy of immobile muscles.

## References

[1] Bodine SC (2013). Disuse-induced muscle wasting. Int J Biochem.

[2] Bonetto A, Aydogdu T, Jin X, Zhang Z, Zhan R, Puzis L, Koniaris LG, Zimmers TA (2012). JAK/STAT3 pathway inhibition blocks skeletal muscle wasting downstream of IL-6 and in experimental cancer cachexia. Am J Physiol.

[3] Chao CY, Yin MC, Huang Cj (2011). Wild bitter gourd extract up-regulates mRNA expression of PPARα, PPARγ and their target genes in C57BL/6J mice. J Ethnopharmacol.

[4] Ching RH, Yeung L O, Tse IM, Sit WH, Li E T (2011). Supplementation of bitter melon to rats fed a high-fructose diet during gestation and lactation ameliorates fructose-induced dyslipidemia and hepatic oxidative stress in male offspring. J Nutr.

[5] Chuang LT, Shih YH, Huang WC, Lin LC, Hsu C, Chyuan JH, Tsai TH, Tsai PJ (2020). In vitro and in vivo screening of wild bitter melon leaf for anti-inflammatory activity against Cutibacterium acnes. Molecules.

[6] Cohen S, Nathan JA, Goldberg AL (2015). Muscle wasting in disease: molecular mechanisms and promising therapies. Nat Rev Drug Discov.

[7] de Oliveira MS, da Costa WA, Bezerra FW F, AraÃºjo ME, Ferreira GC, de Carvalho Junior RN (2018). Phytochemical profile and biological activities of Momordica charantia L (Cucurbitaceae): A review. AJB.

[8] Desaphy JF, Pierno S, Liantonio A, Giannuzzi V, Digennaro C, Dinardo MM, Camerino GM, Ricciuti P, Brocca L, Pellegrino MA (2010). Antioxidant treatment of hindlimb-unloaded mouse counteracts fiber type transition but not atrophy of disused muscles. Pharmacol Res.

[9] Dhar P, Chattopadhyay K, Bhattacharyya D, Roychoudhury A, Biswas A, Ghosh S (2007). Antioxidative effect of conjugated linolenic acid in diabetic and non-diabetic blood: an in vitro study. J Oleo Sci.

[10] Duarte J, Soares J (1997). Supplementation of vitamin E may attenuate skeletal muscle immobilization atrophy. Int J Sports Med.

[11] Fang WY, Tseng YT, Lee TY, Fu YC, Chang WH, Lo WW, Lin CL, Lo YC (2021). Triptolide prevents LPS‐induced skeletal muscle atrophy via inhibiting NF‐κB/TNF‐α and regulating protein synthesis/degradation pathway. Br J Pharmacol.

[12] Farid M, Reid MB, Li YP, Gerken E, Durham WJ (2005). Effects of dietary curcumin or N-acetylcysteine on NF-κB activity and contractile performance in ambulatory and unloaded murine soleus. Nutr Metab (Lond).

[13] Gao Y, Arfat Y, Wang H, Goswami N (2018). Muscle atrophy induced by mechanical unloading: mechanisms and potential countermeasures. Front physiol.

[14] Garrett WE, Mumma M, Lucaveche CL (1983). Ultrastructural differences in human skeletal muscle fiber types. Orthop Clin North Am.

[15] Haddad F, Zaldivar F, Cooper DM, Adams GR (2005). IL-6-induced skeletal muscle atrophy. J Appl Physiol.

[16] Hsu C, Tsai TH, Li YY, Wu WH, Huang CJ, Tsai PJ (2012). Wild bitter melon (Momordica charantia Linn var abbreviata Ser ) extract and its bioactive components suppress Propionibacterium acnes-induced inflammation. Food Chem.

[17] Huang WC, Tsai TH, Huang CJ, Li YY, Chyuan JH, Chuang LT, Tsai PJ (2015). Inhibitory effects of wild bitter melon leaf extract on Propionibacterium acnes-induced skin inflammation in mice and cytokine production in vitro. Food Funct.

[18] Janero DR (1990). Malondialdehyde and thiobarbituric acid-reactivity as diagnostic indices of lipid peroxidation and peroxidative tissue injury. Am J Physiol Cell Physiol.

[19] Judge AR, Koncarevic A, Hunter RB, Liou HC, Jackman RW, Kandarian SC (2007). Role for IκBα, but not c-Rel, in skeletal muscle atrophy. Am J Physiol Cell Physiol.

[20] Koesterer T, Dodd SL, Powers S (2002). Increased antioxidant capacity does not attenuate muscle atrophy caused by unweighting. J Appl Physiol.

[21] Kondo H (1991). Oxidative stress in skeletal muscle atrophied by immobilization. Acta Physiol Scand.

[22] Kumar R, Balaji S, Sripriya R, Nithya N, Uma T, Sehgal P (2010). In vitro evaluation of antioxidants of fruit extract of Momordica charantia L on fibroblasts and keratinocytes. J Agric Food Chem.

[23] Kung WM, Lin CC, Kuo CY, Juin YC, Wu PC, Lin MS (2020). Wild bitter melon exerts anti-inflammatory effects by upregulating injury-attenuated CISD2 expression following spinal cord injury. Behav Neurol.

[24] Kwatra D, Subramaniam D, Ramamoorthy P, Standing D, Moran E, Velayutham R, Mitra A, Umar S, Anant S (2013a). Methanolic extracts of bitter melon inhibit colon cancer stem cells by affecting energy homeostasis and autophagy. Evid Based Complement Alternat Med.

[25] Kwatra D, Venugopal A, Standing D, Ponnurangam S, Dhar A, Mitra A, Anant S (2013b). Bitter melon extracts enhance the activity of chemotherapeutic agents through the modulation of multiple drug resistance. J Pharm Sci.

[26] Lang SM, Kazi AA, Hong-Brown L, Lang CH (2012). Delayed recovery of skeletal muscle mass following hindlimb immobilization in mTOR heterozygous mice. PloS One.

[27] Levine S, Nguyen T, Taylor N, Friscia ME, Budak MT, Rothenberg P, Zhu J, Sachdeva R, Sonnad S, Kaiser LR (2008). Rapid disuse atrophy of diaphragm fibers in mechanically ventilated humans. NEJM.

[28] Ma C, Yu H, Xiao Y, Wang H (2017). Momordica charantia extracts ameliorate insulin resistance by regulating the expression of SOCS-3 and JNK in type 2 diabetes mellitus rats. Pharm Biol.

[29] Nkambo W, Anyama N, Onegi B (2013). In vivo hypoglycemic effect of methanolic fruit extract of Momordica charantia L. Afr Health Sci.

[30] Patwardhan B, Warude D, Pushpangadan P, Bhatt N (2005). Ayurveda and traditional Chinese medicine: a comparative overview. Evid Based Complement Alternat Med.

[31] Powers SK (2014a). Can antioxidants protect against disuse muscle atrophy?. Sports Med.

[32] Powers SK (2014b). Can antioxidants protect against disuse muscle atrophy?. Sports Med.

[33] Psatha M, Wu Z, Gammie FM, Ratkevicius A, Wackerhage H, Lee JH, Redpath TW, Gilbert FJ, Ashcroft GP, Meakin JR (2012). A longitudinal MRI study of muscle atrophy during lower leg immobilization following ankle fracture. J Magn Reson Imaging.

[34] Reid MB, Moylan JS (2011). Beyond atrophy: redox mechanisms of muscle dysfunction in chronic inflammatory disease. Physiol J.

[35] Rommersbach N, Wirth R, Lueg G, Klimek C, Schnatmann M, Liermann D, Janssen G, Müller MJ, Pourhassan M (2020). The impact of disease-related immobilization on thigh muscle mass and strength in older hospitalized patients. BMC Geriatr.

[36] Santos AK, Costa JG, Menezes IR, Cansanção IF, Santos KK, Matias EF, Coutinho HD (2010). Antioxidant activity of five Brazilian plants used as traditional medicines and food in Brazil. Pharmacogn Mag.

[37] Servais S, Letexier D, Favier R, Duchamp C, Desplanches D (2007). Prevention of unloading-induced atrophy by vitamin E supplementation: links between oxidative stress and soleus muscle proteolysis?. Free Radic Biol Med.

[38] Sulaiman SF, Ooi KL (2013). Antioxidant and α-glucosidase inhibitory activities of cucurbit fruit vegetables and identification of active and major constituents from phenolic-rich extracts of Lagenaria siceraria and Sechium edule. J Agric Food Chem.

[39] Thenmozhi AJ, Subramanian P (2011). Antioxidant potential of Momordica charantia in ammonium chloride-induced hyperammonemic rats. Evid Based Complement Alternat Med.

[40] Tripathi UN, Chandra D (2010). Anti-hyperglycemic and anti-oxidative effect of aqueous extract of Momordica charantia pulp and Trigonella foenum graecum seed in alloxan-induced diabetic rats. IJBB.

[41] Van’t Klooster C, van Der Graaf Y, Ridker P, Westerink J, Hjortnaes J, Sluijs I, Asselbergs F, Bots M, Kappelle L, Visseren F (2020). The relation between healthy lifestyle changes and decrease in systemic inflammation in patients with stable cardiovascular disease. Atherosclerosis.

[42] Wang Y, Pessin JE (2013). Mechanisms for fiber-type specificity of skeletal muscle atrophy. Curr Opin Clin Nutr Metab Care.

[43] Xin Sb, Yan H, Ma J, Sun Q, Shen L (2016). Protective effects of luteolin on lipopolysaccharide-induced acute renal injury in mice. Med Sci Monit.

[44] You J-S, Anderson GB, Dooley MS, Hornberger TA (2015). The role of mTOR signaling in the regulation of protein synthesis and muscle mass during immobilization in mice. Dis Model Mech.

